# Multi‐system evaluation of baseline‐referenced gray scale CNR profile deviation for phantom‐based ultrasound quality assurance

**DOI:** 10.1002/acm2.70880

**Published:** 2026-10-27

**Authors:** Ji‐Hye Kim, Yeong‐Cheol Heo

**Affiliations:** ^1^ Department of Radiological Science Chungbuk Health & Science University Cheongju‐si Chungcheongbuk‐do Republic of Korea; ^2^ Department of Radiological Science College of Health Science Eulji University Seongnam‐si Gyeonggi‐do Republic of Korea

**Keywords:** ATS‐539 phantom, baseline‐referenced monitoring, contrast‐to‐noise ratio, transducer surface acoustic shielding, ultrasound quality assurance

## Abstract

**Background:**

Phantom‐based ultrasound quality assurance (QA) commonly incorporates qualitative image assessment, whereas robust baseline‐referenced quantitative indicators for routine monitoring across multiple systems remain limited.

**Purpose:**

To investigate whether controlled transducer surface acoustic shielding produces measurable baseline‐referenced changes in selected dead zone (DZ) and gray scale (GS) indicators across multiple ultrasound systems using an ATS‐539 phantom.

**Methods:**

Four ultrasound systems (GE LOGIQ P10, GE LOGIQ P6, Mindray DC‐70 Exp, and Siemens X150) were evaluated. The transducer aperture was partially shielded with insulating tape at 0%, 5%, 10%, 15%, 20%, and 30% coverage, applied either centrally or at the indicator‐side margin. Five repeated images were acquired for each condition. GS performance was assessed using the contrast‐to‐noise ratio (CNR) of six cylindrical targets. Target‐specific CNR values were converted to within‐device z‐deviations relative to baseline (L0), and the root‐mean‐square z‐deviation across all six targets (RMS‐z) was used as the primary GS profile indicator. DZ performance was evaluated using landmark‐based pin brightness as a secondary near‐field indicator.

**Results:**

Under central shielding, the GS RMS‐z profile deviation score increased with shielding ratio in all four systems (Spearman *ρ* = 0.642–0.975, all *p* < 0.001). Central shielding produced consistent RMS‐z increases across all four systems, whereas indicator‐side shielding yielded a more heterogeneous response: RMS‐z remained positively associated with shielding ratio in three systems (*ρ* = 0.734–0.917, all *p* < 0.001), but the response was weaker and non‐significant in the GE LOGIQ P10. Central DZ pin brightness decreased with increasing shielding ratio across all systems (*ρ* = −0.581 to −0.865, all *p* < 0.001). Baseline CNR profiles and absolute CNR magnitudes differed substantially among systems, and within‐device baseline referencing was therefore preferred over direct cross‐system comparison. Manual landmark placement demonstrated high repeatability for target‐specific CNR and DZ brightness measurements (ICC(A,1) = 0.88–0.97), whereas the aggregate RMS‐z score showed moderate‐to‐good repeatability (ICC(A,1) = 0.771).

**Conclusions:**

Baseline‐referenced GS CNR profile deviation demonstrated a consistent response to controlled acoustic shielding across multiple ultrasound systems. DZ brightness provided complementary near‐field information but remained a secondary indicator. These findings support device‐specific longitudinal monitoring of phantom‐based QA metrics rather than interpretation based on universal cutoff values.

## 1 INTRODUCTION

Ultrasound imaging is widely used in clinical practice because it provides real‐time visualization without exposing patients to ionizing radiation and offers relative portability and affordability compared with many other imaging modalities. Image quality depends not only on system settings but also on the performance and integrity of the transducer. Previous studies have shown that transducer degradation, including defective or inactive elements, can adversely affect image quality through signal loss, image distortion, and reduced measurement accuracy. ^1^, ^2^, ^3^, ^4^ Such degradation is relatively common in routine clinical use and may remain undetected when clinical images are assessed solely by visual inspection.^5^, ^6^, ^7^, ^8^

Maintaining consistent image quality therefore requires routine ultrasound quality assurance (QA) programs capable of detecting gradual changes in system and transducer performance. Long‐term QA initiatives have demonstrated their value in identifying equipment degradation that might otherwise go unnoticed during routine clinical operation.^9^ Accordingly, recommendations from the American Institute of Ultrasound in Medicine and the British Medical Ultrasound Society support periodic QA testing and comparison with baseline or reference images to facilitate longitudinal performance monitoring.^10^, ^11^ AAPM Report 65 and guidance from the American College of Radiology additionally describe technical quality‐control procedures and performance assessment for ultrasound systems.^12^, ^13^ Phantom‐based assessment using multipurpose phantoms such as the ATS‐539 remains a widely adopted approach for evaluating selected QA parameters, including dead zone (DZ), resolution, uniformity, and gray scale (GS) performance.^12^, ^14^, ^15^, ^16^

Despite these recommendations, several commonly used ultrasound QA parameters continue to rely partly on qualitative visual assessment. For example, GS evaluation often depends on an observer's judgment regarding the visibility of cylindrical contrast targets, and previous studies have reported substantial intra‐ and interobserver variability in such assessments.^17^ In addition, the relatively large number of recommended QA tests and their limited integration into routine clinical workflows remain practical barriers to widespread implementation.^18^ Objective, quantitative indicators are therefore needed to complement conventional visual phantom assessment and to support efficient longitudinal monitoring.

Various quantitative and automated approaches have been proposed to improve ultrasound QA. Previous investigations have compared methods for identifying ultrasound image artifacts, developed automated techniques for assessing transducer uniformity using clinical or in‐air images, and applied machine‐learning methods to phantom‐based and clinical QA tasks.^19^, ^20^, ^21^, ^22^, ^23^, ^24^ Quantitative phantom‐based criteria have also been introduced in specific equipment‐assessment and screening settings.^16^, ^25^ However, most previous studies have focused on artifact detection, automated classification, or task‐specific QA applications. Comparatively little evidence is available regarding how selected phantom‐based indicators respond across different ultrasound systems when the degree and location of transducer surface acoustic shielding are systematically controlled—a critical methodological gap, because system‐specific beamforming architectures and aperture geometries may produce substantially different indicator responses under identical perturbation conditions. Understanding such responses is an important step toward establishing baseline‐referenced indicators suitable for device‐specific longitudinal QA monitoring.

To address this gap, we applied controlled transducer surface acoustic shielding using insulating tape positioned either centrally or along the indicator‐side margin of the transducer aperture. Selected DZ and GS indicators were then evaluated across four ultrasound systems using an ATS‐539 phantom. The primary objective was to determine whether a within‐device, baseline‐referenced GS contrast‐to‐noise ratio (CNR) profile metric could quantify shielding‐induced deviations in gray scale performance across systems. DZ pin brightness was evaluated as a complementary near‐field indicator of shielding‐related signal changes. Rather than establishing universal thresholds or assessing clinical defect‐detection performance, this study was designed to characterize the responsiveness of selected QA indicators under controlled perturbation conditions within a device‐specific baseline‐referencing framework.

## 2 MATERIALS AND METHODS

### 2.1 Study design and ethical considerations

This was a phantom‐based, multi‐system experimental study designed to evaluate the responsiveness of selected ultrasound QA indicators to controlled transducer surface acoustic shielding. Four ultrasound systems were evaluated using an ATS‐539 tissue‐mimicking phantom. The primary analysis focused on gray scale (GS) contrast‐to‐noise ratio (CNR) profile deviation, quantified using a within‐device baseline‐referenced root‐mean‐square z‐deviation (RMS‐z) score. Dead zone (DZ) pin brightness was assessed as a secondary near‐field indicator.

All measurements were performed exclusively on a tissue‐mimicking phantom. No human participants, animals, or patient data were used; therefore, the study did not require institutional review board approval. All data analyzed in this study were newly acquired under the present four‐system protocol; in particular, the GE LOGIQ P10 data did not overlap with a previously reported single‐system ATS‐539 shielding study.^26^

### 2.2 Ultrasound systems and phantom

Four diagnostic ultrasound systems from three vendors (GE HealthCare, Mindray, and Siemens Healthineers) were evaluated: GE LOGIQ P10, GE LOGIQ P6, Mindray DC‐70 Exp (hereafter DC‐70), and Siemens X150. The following convex transducers were used: the C1‐5 for both GE systems, the SC6‐1E for the Mindray DC‐70 Exp, and the CH5‐2 for the Siemens X150 (Table 1).

**TABLE 1:** Ultrasound systems, transducers, and acquisition settings.

Parameter	GE LOGIQ P10	GE LOGIQ P6	Mindray DC‐70 Exp	Siemens X150
Transducer	C1‐5	C1‐5	SC6‐1E	CH5‐2
Preset	Abdominal	Abdominal	Abdominal	Abdominal
Frequency	4.0 MHz	4.0 MHz	1.9–4.6 MHz	3.6 MHz
Gain	60	60	60	35
Dynamic range	69 dB	69 dB	120 dB	70 dB
Acoustic output	100%	100%	100%	100%
Depth (DZ/GS)	5/14 cm	5/14 cm	5/14 cm	5/14 cm
Software version	R4.5.8	R3.0.8	4.5.0	2.5.03 (1237)

*Note*: Harmonic imaging, spatial compound imaging, speckle reduction, edge enhancement, and vendor‐specific automatic image‐optimization functions, including Auto Tissue Optimization (ATO) or equivalent functions, were disabled for all acquisitions.

All measurements were performed using an ATS‐539 multipurpose ultrasound phantom (ATS Laboratories–CIRS, Norfolk, VA, USA). The phantom consists of a urethane‐rubber tissue‐mimicking material with a speed of sound of 1450 m/s and an attenuation coefficient of 0.5 dB/cm/MHz. For the present analysis, the DZ pin targets (**near**‐field group at 2.0–10.0 mm depth in 1.0 mm increments) and the six GS cylindrical targets (contrast levels of +15, +6, +3, −3, −6, and −15 dB relative to background, located at 4.0 cm depth) were used to derive the selected quantitative QA indicators. Other phantom structures were not analyzed.

### 2.3 Shielding model

A related surface‐occlusion approach using polyvinyl chloride tape has previously been used to simulate failed transducer elements in ultrasound quality‐control testing.^27^ In the present study, controlled transducer surface acoustic shielding was applied using an aperture‐occlusion method, in which vinyl electrical insulating tape (Scotch Super 33+, 3 M, St. Paul, MN, USA; polyvinyl chloride backing, 0.18 mm thick) was placed directly on the transducer surface to partially cover the active aperture. The shielding ratio was defined as the proportion of the active aperture length covered along the long axis of the probe.

Six shielding levels were used: 0%, 5%, 10%, 15%, 20%, and 30%. The occlusion length was scaled to the active aperture of each probe; for example, for the 70‐mm GE convex probes, the corresponding occlusion lengths were 0, 3.5, 7.0, 10.5, 14.0, and 21.0 mm. Shielding was applied at two aperture positions: the central region (C) and the indicator‐side margin (M). The indicator‐side margin was defined as the side of the active aperture corresponding to the transducer orientation marker; in the displayed DZ images, this side corresponded to pins 1–3 (Figure 1b,d,e). Central shielding represented symmetric aperture occlusion, whereas indicator‐side shielding represented asymmetric peripheral occlusion. The 0% condition served as the baseline (L0), and the remaining conditions consisted of five central shielding levels (L1_C–L5_C) and five indicator‐side shielding levels (L1_M–L5_M). Five repeated images were acquired for each condition, yielding 55 images per system. Representative photographs and schematic illustrations of the central and indicator‐side shielding configurations are shown in Figure 1c–e.

**FIGURE 1:**
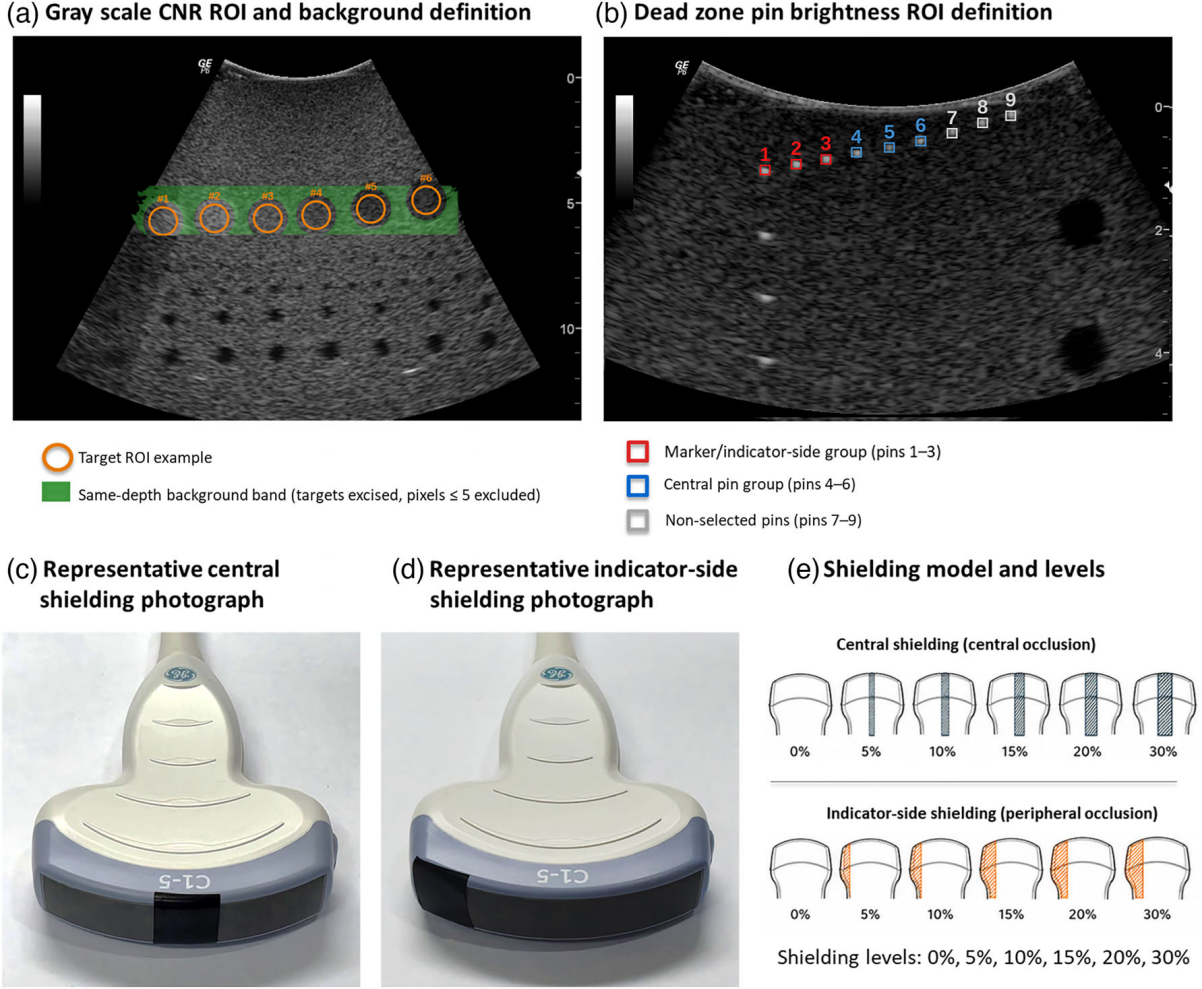
Experimental setup, ROI definition, and transducer surface shielding model. (a) Gray scale CNR measurement. Representative GE LOGIQ P6 image showing circular ROIs placed on the six ATS‐539 gray scale targets and a same‐depth background region used for CNR calculation after excluding target ROIs with a 10‐pixel margin and pixels with intensity ≤5. (b) Dead zone pin brightness measurement. Representative GE LOGIQ P6 image showing 13 × 13 pixel ROIs centered on the nine DZ pins. Pins 1–3 were used for indicator‐side shielding analysis, and pins 4–6 were used for central shielding analysis. (c) Representative central transducer surface shielding. (d) Representative indicator‐side transducer surface shielding. (e) Schematic illustration of the central and indicator‐side shielding model. Shielding was applied at 0%, 5%, 10%, 15%, 20%, and 30% of the active aperture length.

### 2.4 Image acquisition

All images were acquired using standardized settings within each ultrasound system (Table 1) to reduce the influence of image‐processing variability. All phantom images were acquired by a single operator using a consistent probe‐positioning, coupling, and acquisition procedure within each ultrasound system. Harmonic imaging, spatial compound imaging, speckle reduction, and edge enhancement were disabled for all acquisitions. Vendor‐specific automatic image‐optimization functions, including Auto Tissue Optimization (ATO) or equivalent functions, were not used during image acquisition. Images were acquired using a user‐defined preset derived from the factory abdominal preset of each system, in which the focal position and gain were adjusted once by the operator for clear visualization of the phantom targets and then kept fixed. Transmit power (acoustic output) was set to 100% for all systems, and TGC controls were kept at the centered (flat) position. The grayscale map was kept unchanged within each system throughout all shielding conditions. Images were exported from each system in DICOM format, opened in ImageJ, and saved as uncompressed TIFF files without resampling or intensity rescaling; all measurements were performed on these TIFF images on the original pixel grid with no further image processing. The analyzed data were therefore vendor‐processed B‐mode images rather than raw RF data; because the processing chain was identical across all shielding conditions within each system, shielding‐induced changes were evaluated relative to a baseline acquired under the same processing.

Within each system, imaging depth differed between the DZ (5 cm) and GS (14 cm) acquisitions to match the respective target locations, whereas all other settings (gain, dynamic range, and frequency) were identical between the two acquisitions and were maintained across all shielding conditions.

### 2.5 Gray scale measurement

GS assessment was performed using the six cylindrical targets of the ATS‐539 phantom, with nominal contrasts of +15, +6, +3, −3, −6, and −15 dB. Target centers were manually identified in ImageJ using the Multi‐point tool on the original pixel grid. Circular regions of interest (ROIs) were placed at each target center, using device‐specific radii of 22 pixels for both GE systems, 30 pixels for the Mindray DC‐70 Exp, and 15 pixels for the Siemens X150. Owing to differences in display pixel spacing, these pixel radii corresponded to physical radii of 5.31 mm (GE LOGIQ P10), 7.24 mm (GE LOGIQ P6), 6.79 mm (Mindray DC‐70 Exp), and 4.57 mm (Siemens X150). The ROI radius was selected separately for each system to allow reproducible placement within the visible cylindrical target boundary on the native image grid while minimizing inclusion of the surrounding background. Because display pixel spacing and target representation differed among systems, applying a single pixel radius or a single physical radius across all systems would not have provided equivalent target sampling. Within each system, however, the same ROI radius was maintained for all six targets and all shielding conditions. Differences in ROI size may have contributed to differences in absolute CNR magnitude; therefore, raw CNR values were presented descriptively and were not interpreted on a common cross‐system scale. The primary analysis used within‐device baseline‐referenced z‐deviations.

For each image, the background was defined as a same‐depth representative region. This region consisted of a horizontal band spanning the depth range of the six GS targets, excluding all target ROIs with a 10‐pixel margin and excluding pixels with an intensity of 5 or below to avoid sector‐boundary contamination. A single background estimate was used for all six targets within each image. Representative GS target ROI placement and the same‐depth background‐region definition are illustrated in Figure 1a.

CNR was calculated for each target as:

$$CNR_{i}=\frac{\left|\mu_{t_{i}}-\;\mu_{bg}\right|}{\sigma_{bg}}$$

where $\mu_{t_{i}}$ is the mean gray level within the target ROI, $\mu_{bg}$ is the mean gray level of the background region, and $\sigma_{bg}$ is the background standard deviation.

For each system and target, baseline CNR was defined from the five L0 acquisitions. Target‐level z‐deviation was then calculated as:

$$z_{i}=\frac{CNR_{i}-\mu_{L0,i}}{\sigma_{L0,i}}$$

where $\mu_{L0,i}$ and $\sigma_{L0,i}$ are the mean and standard deviation of the baseline CNR values for the same system and target. This within‐device normalization expresses each target's CNR change relative to its own baseline variability. Signed z‐deviation refers to the target‐level z‐score with its algebraic sign retained. A positive signed z‐deviation indicates that the target CNR is higher than the corresponding device‐ and target‐specific baseline mean, whereas a negative value indicates that it is lower than the baseline mean. The resulting six target‐level z‐deviations were used to construct the GS CNR profile metrics described in Section 2.7.

### 2.6 Dead zone measurement

Dead zone assessment was performed using landmark‐based manual measurement in ImageJ (National Institutes of Health, Bethesda, MD, USA). For each DZ image, the centers of the nine dead zone pins were identified on the original pixel grid using the Multi‐point tool, with pins marked from left to right.

Pin brightness was defined as the mean pixel intensity within a fixed square region of interest (ROI) measuring 13 × 13 pixels, centered on each pin. Pin groups were matched to the shielding position: the central pin group (pins 4–6) was used for central shielding, and the indicator‐side pin group (pins 1–3) for indicator‐side shielding. Representative DZ pin numbering, ROI placement, and pin‐group definitions are illustrated in Figure 1b. The unweighted nine‐pin mean was not used, because shielding was applied to a localized aperture region and averaging across perturbed and unperturbed pins would obscure the position‐specific response. Distance‐based DZ measurement was not retained as a final outcome after method‐feasibility assessment because it did not provide a reproducible or position‐specific response suitable for the present shielding model. The final DZ analysis therefore focused on pin brightness as a secondary near‐field indicator of shielding‐related signal change.

### 2.7 Statistical analysis and indicator hierarchy

All analyses were performed using Python (version 3.14.4) with NumPy (version 2.4.4), SciPy (version 1.17.1), and pandas (version 3.0.2). Intraclass correlation coefficients were computed using the pingouin library (version 0.6.1). A hierarchical indicator framework was used to avoid overinterpreting single‐target or single‐parameter responses.

The primary indicator was the GS RMS‐z profile deviation score:

$$\mathrm{RMS}-z\;=\sqrt{\frac{1}{6}\sum_{i=1}^{6}{\left(z_{i}\right)}^{2}}$$

where $z_{i}$ represents the target‐specific CNR z‐deviation for each of the six GS targets. RMS‐z quantified the overall deviation of the GS CNR profile from the device‐specific baseline.

For the primary RMS‐z indicator, Spearman's rank correlation coefficient (*ρ*) was used to evaluate the monotonic association between shielding ratio and profile deviation under central and indicator‐side shielding. The coefficient ranges from −1 to +1; positive ρ values indicated that RMS‐z tended to increase with shielding ratio, whereas absolute values closer to 1 indicated a stronger monotonic association.

DZ pin brightness was treated as a secondary near‐field indicator. For the DZ analysis, Spearman rank correlation was computed between shielding ratio and pin‐group brightness. All tests were two‐sided, with *α* = 0.05.

Measurement repeatability was evaluated using the newly acquired GE LOGIQ P10 dataset. The same observer repeated landmark placement on the same images in two independent sessions without reference to the first‐session coordinates. ICC(A,1)—a two‐way random‐effects, absolute‐agreement, single‐measure intraclass correlation coefficient—was calculated for DZ pin brightness, image‐level DZ summary measures, target‐specific GS CNR values, and the per‐image GS RMS‐z profile deviation score.

## 3 RESULTS

### 3.1 Gray scale indicators

Baseline CNR profiles differed across systems. At L0, the six‐target GS CNR profiles showed a similar overall pattern, but the absolute CNR magnitude differed among the four ultrasound systems (Figure 2).

**FIGURE 2:**
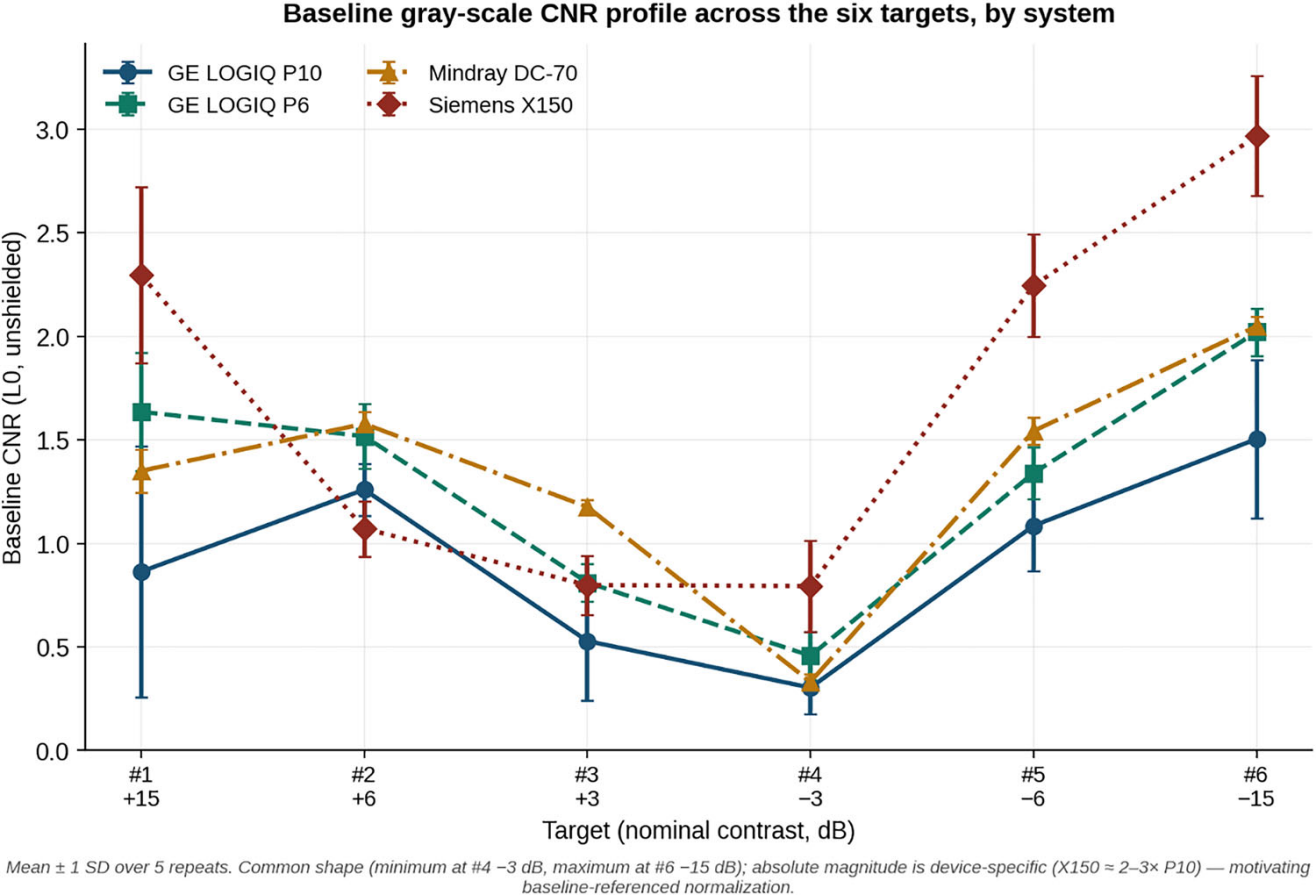
Baseline gray scale contrast‐to‐noise ratio profiles. Each curve represents the mean CNR from five baseline (L0) acquisitions across the six ATS‐539 gray scale targets, ordered by nominal contrast from +15 dB (target #1) to −15 dB (target #6). All systems showed a V‐shaped baseline profile, with the lowest CNR observed at target #4 (−3 dB). However, the absolute CNR magnitude differed across systems; within‐device baseline‐referenced z‐deviation was therefore used instead of direct cross‐system comparison of raw CNR values.

The primary GS indicator was the RMS‐z profile deviation score, which quantified the overall deviation of the six‐target CNR profile from each system's own baseline. Under central shielding, RMS‐z increased with shielding ratio in all four systems, with Spearman's *ρ* values of +0.642 for the GE LOGIQ P10, +0.975 for the GE LOGIQ P6, +0.930 for the Mindray DC‐70 Exp, and +0.971 for the Siemens X150 (all *p* < 0.001; Table 2, Figure 3). Although individual shielding levels did not increase strictly monotonically in every system, notable non‐monotonic steps were observed in the GE LOGIQ P10 under central shielding (see Figure 3, top‐left panel); the overall rank correlation between shielding ratio and RMS‐z was nevertheless consistently positive across all systems. Mean RMS‐z increased from baseline to 30% central shielding in every system.

**TABLE 2:** Primary GS RMS‐z profile deviation by shielding position.

System	Shielding position	Spearman *ρ*	*p* value	Summary interpretation
GE LOGIQ P10	Central	0.642	<0.001	Positive RMS‐z response
GE LOGIQ P6	Central	0.975	<0.001	Positive RMS‐z response
Mindray DC‐70 Exp	Central	0.930	<0.001	Positive RMS‐z response
Siemens X150	Central	0.971	<0.001	Positive RMS‐z response
GE LOGIQ P10	Indicator‐side	0.263	0.160	Weaker, non‐significant response
GE LOGIQ P6	Indicator‐side	0.917	<0.001	Positive RMS‐z response
Mindray DC‐70 Exp	Indicator‐side	0.734	<0.001	Positive RMS‐z response
Siemens X150	Indicator‐side	0.743	<0.001	Positive RMS‐z response

*Note*: RMS‐z was calculated across all six GS targets relative to the corresponding device‐specific baseline.

**FIGURE 3:**
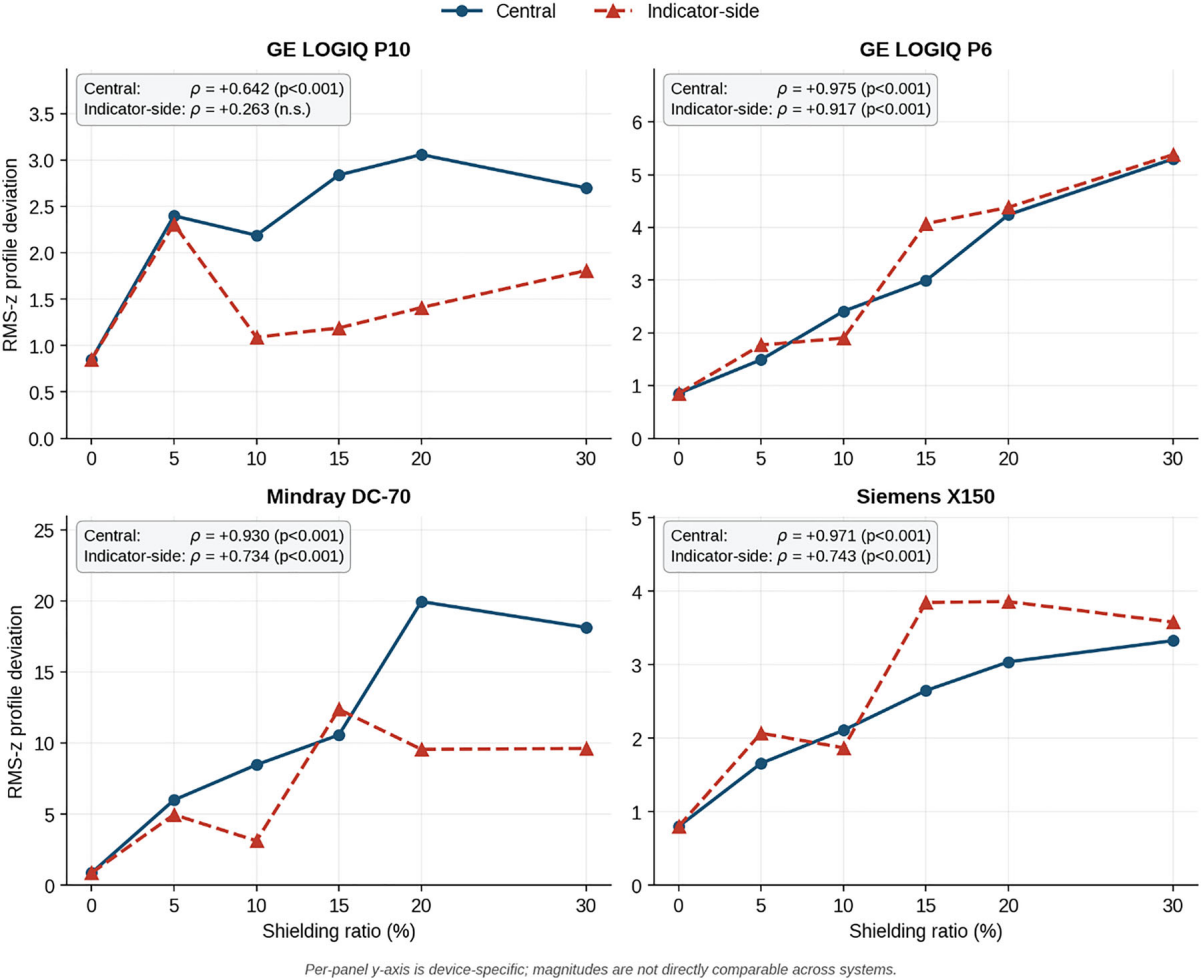
Primary gray scale indicator: RMS‐z profile deviation by shielding ratio. Root‐mean‐square z‐deviation (RMS‐z) of the gray scale CNR profile plotted against shielding ratio for each ultrasound system. RMS‐z was calculated from target‐specific z‐deviations relative to each system's own baseline (L0) and was used as the primary gray scale profile indicator. Solid lines indicate central shielding, and dashed lines indicate indicator‐side shielding. Spearman rank correlation coefficients (*ρ*) between shielding ratio and RMS‐z are shown within each panel. Under central shielding, RMS‐z increased with shielding ratio in all four systems. Under indicator‐side shielding, the response was position‐specific and weaker in the GE LOGIQ P10. Each panel uses a device‐specific y‐axis scale to emphasize within‐system trends; therefore, RMS‐z magnitudes should not be visually compared directly across systems.

Under indicator‐side shielding, RMS‐z also showed a positive association with shielding ratio in three systems: GE LOGIQ P6 (*ρ* = +0.917), Mindray DC‐70 Exp (*ρ* = +0.734), and Siemens X150 (*ρ* = +0.743), all *p* < 0.001. The GE LOGIQ P10 showed a weaker, non‐significant trend (*ρ* = +0.263, *p* = 0.160). These findings indicate that the RMS‐z profile metric responded to shielding at both aperture positions, although the magnitude of the response depended on shielding position and system.

The secondary profile‐shape analysis was based on the signed‐z heatmap (Figure 4). Under central shielding, the signed‐z profile showed structured target‐level deviations across systems. In particular, the two targets adjacent to the 0 dB boundary deviated in opposite directions: target #3 (+3 dB) negatively and target #4 (−3 dB) positively. Under indicator‐side shielding, the signed‐z pattern differed from the central shielding pattern, consistent with a position‐specific profile‐shape response.

**FIGURE 4:**
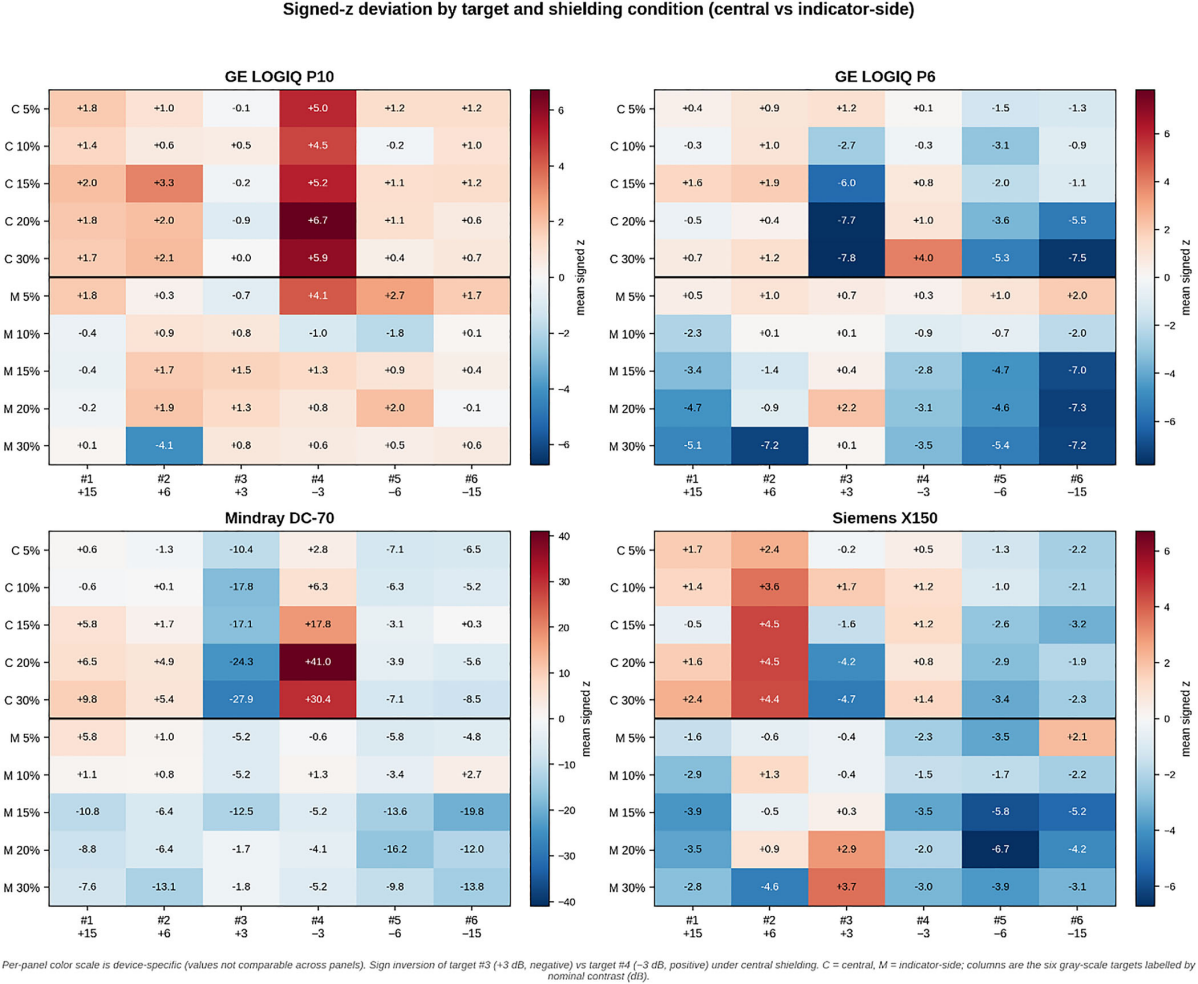
Secondary profile‐shape analysis: signed z‐deviation heatmap. Signed z‐deviation heatmap of the gray scale CNR profile across shielding ratio and target position. Each cell represents the signed z‐deviation of a single gray scale target relative to the corresponding device‐ and target‐specific baseline. Rows indicate shielding condition under central shielding (C) and indicator‐side shielding (M), and columns indicate the six ATS‐539 gray scale targets ordered from +15 dB (target #1) to −15 dB (target #6). In the color version, positive deviations are shown in red and negative deviations in blue, with a per‐panel scale centered at zero. Because scaling is applied within each device, color represents the direction and relative magnitude of deviation within a system rather than absolute comparability across systems. Under central shielding, the near‐boundary targets showed an opposing signed‐z pattern, with target #3 (+3 dB) tending toward negative deviation and target #4 (−3 dB) toward positive deviation. Under indicator‐side shielding, the profile‐shape pattern differed from the central shielding pattern.

### 3.2 Dead zone indicators

Under central shielding, the mean brightness of the central pin group decreased with increasing shielding ratio in all four systems (Table 3): GE LOGIQ P10 (*ρ* = −0.581), GE LOGIQ P6 (*ρ* = −0.865), Mindray DC‐70 Exp (*ρ* = −0.705), and Siemens X150 (*ρ* = −0.854), all *p* < 0.001. Central DZ pin brightness thus provided complementary near‐field information on shielding‐related signal attenuation. Under indicator‐side shielding, the indicator‐side pin group showed a weaker and less consistent response. Indicator‐side pin brightness was significantly negatively correlated with shielding ratio only in the Siemens X150 (*ρ* = −0.531, *p* = 0.003); the correlation did not reach statistical significance in the GE LOGIQ P6 (*ρ* = −0.326, *p* = 0.079) or the GE LOGIQ P10 (ρ = −0.267, *p* = 0.153), and the Mindray DC‐70 Exp showed a positive correlation (*ρ* = +0.389, *p* = 0.034). Because the direction and magnitude of the indicator‐side DZ response varied across systems, this result was interpreted as descriptive position‐specific information rather than as a retained DZ indicator.

**TABLE 3:** Secondary DZ pin brightness response by shielding position.

System	Shielding position	Pin group	Spearman *ρ*	*p* value	Summary interpretation
GE LOGIQ P10	Central	Pins 4–6	−0.581	<0.001	Decreased with shielding
GE LOGIQ P6	Central	Pins 4–6	−0.865	<0.001	Decreased with shielding
Mindray DC‐70 Exp	Central	Pins 4–6	−0.705	<0.001	Decreased with shielding
Siemens X150	Central	Pins 4–6	−0.854	<0.001	Decreased with shielding
GE LOGIQ P10	Indicator‐side	Pins 1–3	−0.267	0.153	Non‐significant response
GE LOGIQ P6	Indicator‐side	Pins 1–3	−0.326	0.079	Negative, non‐significant trend
Mindray DC‐70 Exp	Indicator‐side	Pins 1–3	+0.389	0.034	Direction differed from other systems
Siemens X150	Indicator‐side	Pins 1–3	−0.531	0.003	Weaker position‐specific response

*Note*: DZ pin brightness was interpreted as a complementary secondary near‐field indicator. Indicator‐side DZ findings were interpreted descriptively because the direction and magnitude of response varied across systems.

### 3.3 Repeatability

Intra‐observer repeatability of landmark‐based measurements was evaluated using the newly acquired GE LOGIQ P10 dataset (Table 4). For the GS analysis, target‐specific CNR measurements showed high repeatability, with ICC(A,1) values ranging from 0.877 to 0.965 across the six GS targets. The overall CNR summary also showed high repeatability (ICC(A,1) = 0.971). The aggregate RMS‐z profile deviation score showed moderate‐to‐good repeatability (ICC(A,1) = 0.771). This lower value relative to the underlying CNR measurements likely reflects the effect of baseline standard‐deviation normalization, in which small landmark‐related CNR differences can be amplified for targets with low baseline variability. For the DZ analysis, ICC(A,1) was 0.925 at the pin level and 0.972 for the central pin‐group image summary. The indicator‐side pin‐group summary showed a comparable ICC(A,1) of 0.950. Overall, the manual landmarking procedure yielded repeatable GS CNR and DZ brightness measurements, whereas the RMS‐z score was more sensitive to small differences introduced during landmark placement.

**TABLE 4:** Intra‐observer repeatability of landmark‐based measurements.

Analysis	Measurement	ICC(A,1)	Interpretation
GS	Target‐specific CNR	0.877–0.965	High repeatability
GS	Overall CNR summary	0.971	High repeatability
GS	RMS‐z profile deviation score	0.771	Moderate‐to‐good repeatability
DZ	Pin‐level brightness	0.925	High repeatability
DZ	Central pin‐group image summary	0.972	High repeatability
DZ	Indicator‐side pin‐group image summary	0.950	High repeatability

*Note*: ICC(A,1) indicates a two‐way random‐effects, absolute‐agreement, single‐measure intraclass correlation coefficient. Repeatability was evaluated using the newly acquired GE LOGIQ P10 dataset.

## 4 DISCUSSION

This study examined the behavior of baseline‐referenced GS and DZ indicators under controlled transducer surface acoustic shielding across four ultrasound systems. The most consistent finding was the increase in the GS CNR‐based RMS‐z profile deviation score with increasing central shielding. Because RMS‐z incorporates information from all six GS targets relative to each system's own baseline, it captures overall profile disruption rather than isolated target‐specific changes. The consistent response observed across all systems suggests that baseline‐referenced GS CNR profile analysis may provide a useful quantitative framework for longitudinal phantom‐based QA monitoring. Absolute CNR values also varied substantially among systems, even under baseline conditions. This observation is consistent with previous reports demonstrating system‐dependent differences in low‐contrast detectability when assessed using grayscale phantoms.^28^ Raw CNR values therefore cannot be interpreted reliably on a common cross‐system scale. Instead, each system requires its own baseline GS CNR profile against which subsequent QA measurements can be compared. This device‐specific baseline‐referencing approach is consistent with established QA practice, in which acceptance‐test or baseline images are used as references for longitudinal monitoring rather than as universal pass/fail thresholds.^10^, ^11^, ^29^ Although major acquisition settings and image‐processing options were held constant across shielding conditions within each system, their absolute values and vendor‐specific implementations were not harmonized across systems. Differences in gain mapping, dynamic‐range compression, frequency‐dependent beamforming, aperture weighting, and proprietary image processing may therefore have contributed to between‐system differences in baseline values and response magnitudes. Automatic image‐optimization functions were not used, but residual platform‐specific processing could not be eliminated. This consideration further supports interpreting the primary GS findings within a device‐specific baseline‐referencing framework rather than making direct cross‐system comparisons of absolute values.

In contrast to the GS profile metric, DZ pin brightness primarily reflected localized changes in near‐field signal intensity. Central pin‐group brightness decreased consistently with increasing shielding across all four systems, indicating sensitivity to shielding‐related attenuation of acoustic transmission. By comparison, indicator‐side responses were less uniform and varied among systems, suggesting a greater influence of system‐specific imaging characteristics and aperture geometry. Although DZ brightness provided useful complementary information regarding near‐field behavior, its performance was less consistent than that of the GS RMS‐z profile metric and was therefore retained as a secondary indicator within the proposed QA framework. Recent advances in ultrasound QA have increasingly focused on automated image analysis, artifact detection, and machine‐learning‐based assessment strategies.^18^, ^19^, ^20^, ^21^, ^22^, ^23^, ^24^ Rather than developing a classification algorithm, the present study provides experimental evidence that selected phantom‐based indicators — particularly the GS CNR profile deviation metric — respond consistently to controlled aperture perturbations across multiple ultrasound systems. This controlled‐response framework may provide a methodological basis for future automated QA approaches.

Comparison of central and indicator‐side shielding demonstrated that the RMS‐z profile metric was responsive to aperture occlusion at both locations, although the magnitude and pattern of response varied among systems. Central shielding produced the most consistent increase in RMS‐z, whereas indicator‐side shielding generated a more heterogeneous response. This difference may reflect the asymmetric nature of peripheral aperture occlusion and its interaction with system‐specific aperture weighting, beamforming, or image‐processing characteristics. The GE LOGIQ P10 exhibited the weakest response under indicator‐side shielding, with no significant association between RMS‐z and shielding ratio. However, the mechanism underlying this system‐specific response was not directly evaluated, and the present data cannot distinguish among these potential explanations. Further studies incorporating system‐specific technical characterization are needed to determine the basis of this response.

The signed‐z heatmap provided complementary information on how the GS CNR profile changed across targets. In contrast to RMS‐z, which summarizes the overall magnitude of profile deviation, signed‐z analysis preserves the direction of target‐level changes. In the present study, central and indicator‐side shielding produced different signed‐z patterns, indicating that similar RMS‐z increases may arise from different profile‐shape changes. Therefore, the signed‐z heatmap is best interpreted as a secondary profile‐shape tool that helps contextualize the primary RMS‐z result, rather than as an independent decision metric.

The proposed baseline‐referenced framework also has practical implications for routine ultrasound QA programs. By transforming the six‐target GS CNR profile into a device‐specific RMS‐z deviation score, the present approach offers a quantitative method for monitoring departures from an individual system's established baseline.

Further work should evaluate long‐term baseline stability to determine the broader applicability of these indicators in routine QA practice.

### 4.1 Limitations

First, the shielding model represented a controlled experimental perturbation rather than a direct simulation of clinically occurring transducer failure. Although insulating‐tape occlusion allowed systematic control of shielding location and extent, it does not reproduce the full spectrum of failure mechanisms encountered in clinical practice, including element dropout, electrical faults, phase mismatch, or matching‐layer damage. Accordingly, the present findings should be interpreted as evidence of indicator responsiveness under controlled conditions rather than as a measure of clinical defect‐detection performance. Clinically defective transducers were not included in the present study; therefore, validation of the proposed indicators in probes with confirmed clinical defects remains necessary.

Second, the generalizability of the findings remains limited. Although four ultrasound systems from three manufacturers were included, all measurements were obtained using convex transducers and a single phantom model. Moreover, shielding was applied only along the lateral aperture direction, and elevational shielding was not evaluated. In addition, response patterns under indicator‐side shielding were more variable across systems, suggesting that system‐specific imaging characteristics may influence the observed profile behavior. Further studies incorporating additional probe types, imaging presets, phantom models, and clinical QA environments are needed to determine the broader applicability of the proposed indicators.

Third, several measurement‐related limitations should be acknowledged. Baseline normalization was derived from only five unshielded acquisitions. Because σ_L0 is estimated from this small sample, the baseline variance estimate may be imprecise, potentially leading to inflated z‐scores for targets with inherently low within‐session CNR variability and attenuated z‐scores for targets with high variability. Future studies should use larger baseline sample sizes to improve the stability of baseline variance estimates. Furthermore, the analysis relied on manual landmark placement. Although intra‐observer repeatability was generally high, inter‐observer reproducibility was not evaluated. Users with different landmark‐placement conventions may produce systematically different RMS‐z scores due to propagation of inter‐observer CNR differences through the baseline normalization step. Automated target localization may further improve measurement robustness and facilitate broader implementation of the proposed approach. Additionally, all phantom images were acquired by a single operator; therefore, acquisition‐related inter‐operator variability was not evaluated. In routine QA, probe positioning, contact pressure, phantom alignment, and acoustic coupling may influence CNR and DZ brightness measurements.

## 5 CONCLUSIONS

In this multi‐system phantom study, the baseline‐referenced GS CNR profile deviation metric (RMS‐z) demonstrated a consistent response to controlled transducer surface acoustic shielding across four ultrasound platforms. DZ pin brightness added complementary near‐field information but showed greater position‐ and system‐dependent variability. The observed differences in baseline CNR profiles and response magnitudes further support the use of device‐specific reference data rather than universal thresholds for phantom‐based QA. Taken together, these findings support baseline‐referenced quantitative QA strategies and warrant further validation in longitudinal monitoring and clinically defective transducers.

## AUTHOR CONTRIBUTIONS

**Ji‐Hye Kim**: Conceptualization; methodology; investigation; data curation; writing—original draft. **Yeong‐Cheol Heo**: Conceptualization; supervision; funding acquisition; writing—review & editing.

## CONFLICT OF INTEREST STATEMENT

The authors declare no conflicts of interest.

## ETHICS STATEMENT

This study used a tissue‐mimicking phantom and did not involve human subjects or animals; institutional review board approval was not required.

## Data Availability

The data that support the findings of this study are available from the corresponding author upon reasonable request.
